# Coastal regions of the northern Antarctic Peninsula are key for gentoo populations

**DOI:** 10.1098/rsbl.2020.0708

**Published:** 2021-01-27

**Authors:** Malgorzata Korczak-Abshire, Jefferson T. Hinke, Gennadi Milinevsky, Mariana A. Juáres, George M. Watters

**Affiliations:** 1 Institute of Biochemistry and Biophysics, Polish Academy of Sciences, 02-106 Warsaw, Poland; 2 Antarctic Ecosystem Research Division, Southwest Fisheries Science Center, National Marine Fisheries Service, National Oceanic and Atmospheric Administration, La Jolla, CA 92037, USA; 3 Department of Atmospheric Physics and Geospace, National Antarctic Scientific Center of Ukraine, Kyiv 01601, Ukraine; 4 Physics Faculty, Taras Shevchenko National University of Kyiv, Kyiv 01033, Ukraine; 5 Departamento Biología de Predadores Tope, Instituto Antártico Argentino, San Martín, Buenos Aires B1650CSP, Argentina; 6 National Scientific and Technical Research Council (CONICET), Ciudad Autónoma de Buenos Aires, C1425FQB, Argentina

**Keywords:** *Pygoscelis papua*, climate change, range expansion, satellite telemetry

## Abstract

Southern Ocean ecosystems are rapidly changing due to climate variability. An apparent beneficiary of such change in the western Antarctic Peninsula (WAP) is the gentoo penguin *Pygoscelis papua*, which has increased its population size and expanded its range southward in the last 20 years. To better understand how this species has responded to large-scale changes, we tracked individuals during the non-breeding winter period from five colonies across the latitudinal range of breeding sites in the WAP, including from a recently established colony. Results highlight latitudinal gradients in movement; strong associations with shallow, coastal habitats along the entire Antarctic Peninsula; and movements that are independent of, yet constrained by, sea ice. It is clear that coastal habitats essential to gentoo penguins during the breeding season are similarly critical during winter. Larger movements of birds from northern colonies in the WAP further suggest that leap-frog migration may influence colonization events by facilitating nest-area prospecting and use of new haul-out sites. Our results support efforts to develop a marine protected area around the WAP. Winter habitats used by gentoo penguins outline high priority areas for improving the management of the spatio-temporally concentrated krill (*Euphausia superba*) fishery that operates in this region during winter.

## 1. Introduction

Climate change fundamentally alters the structure and function of marine ecosystems by modifying ocean productivity, altering food-web dynamics and shifting species distributions [1]. Polar ecosystems are especially sensitive to climate perturbations because they are largely structured by the seasonal dynamics of sea ice [2], which have exhibited trends in extent and duration in both hemispheres [3]. An area of particular concern is the western Antarctic Peninsula (WAP), where increases in air and sea-surface temperatures over the last 40 years have reduced regional sea ice extent and duration [4]. Such physical perturbations are associated with changes in the distribution, abundance and survival of several species in the WAP ecosystem [5]. Further compounding risk to this ecosystem is the expanding fishery for Antarctic krill (*Euphausia superba*) [6,7], the largest, by mass, in the Southern Ocean [8].

Seabirds are important indicators of ecosystem status and are among the species impacted by climate change and fisheries [7,9–11]. Changes in population sizes and phenologies due to environmental variation in the Southern Hemisphere are evident [9,12–14]. For example, in the WAP, populations of ice-dependent Adélie penguins (*Pygoscelis adeliae*) and ice-tolerant chinstrap penguins (*P. antarcticus*) have declined [12,15], while the abundance and range of ice-avoiding gentoo penguins (*P. papua*) have increased [12]. Notably, range expansion and rapid population growth of gentoo penguins is occurring at the southern margin of their breeding range, where at least seven newly established colonies have been identified in last 20 years (electronic supplementary material, figure S1) [16]. Despite divergent population trends among the pygoscelid penguins, all three species have been affected by recent krill fishing during the non-breeding period [7], hereafter winter. Thus, although generally considered to be climate ‘winners’ [17], the risks to gentoo penguin populations should be further assessed to better inform conservation and management actions.

Across the WAP, gentoo penguins typically forage within 20 km of breeding sites during the austral summer [18–22]. During winter, gentoos are not constrained by the need to provision chicks and can undertake longer range movements. Prior tracking studies from the South Shetland Islands [18,21] suggested winter movements up to 10 times farther than during summer. Such dispersal to distant foraging areas is the primary mechanism by which range expansion could occur [23], but a lack of tracking data from colonies throughout the WAP limits understanding of how this seabird distributes during the winter and whether there is variation in movement among colonies. Given rapidly changing environmental and anthropogenic drivers in the WAP, identifying winter movements and patterns of habitat use by gentoo penguins are also useful for assessing population status and risks to the species. We therefore tracked the winter movements of gentoo penguins from five colonies across the latitudinal range of this species in the WAP, including from a recently established colony [24] near the southern limit of the species' range.

## 2. Material and methods

We tracked 10 fledgling and 65 post-moult adult gentoo penguins from five colonies across the latitudinal range of breeding colonies in the WAP from February 2017 through January 2018 (table 1; electronic supplementary material, figure S1*c*). We used Sirtrack Kiwisat-202K2G-172A satellite transmitters (60 × 27 × 17 mm, 34 g) to track fledglings and Wildlife Computers Spot-275 satellite transmitters (86 × 17 × 18 mm, 38 g) to track adults. All birds were captured on beaches and the transmitters were affixed to back feathers using glue and cable ties [25]. Transmitters were scheduled to transmit daily from 12:00 to 18:00 UTC, corresponding to daylight hours when birds should be foraging.

**Table 1.** Tagging locations and mean deployment durations (range in parentheses). Tagging locations were at Lions Rump (LRP), Stranger Point (SPS), Cape Shirreff (CAS), Cierva Cove (CVA) and the Argentine Islands (AIS). All tags were released between 4 February and 29 March 2017.

age class	colony	longitude	latitude	*N*	duration (d)	maximum distance (km)
adult	LRP	−58.13	−62.14	15	105 (28–148)	109 (32–229)
	SPS	−58.62	−62.27	13	79 (45–157)	128 (29–186)
	CAS	−60.80	−62.46	9	100 (45–194)	151 (27–251)
	CVA	−60.98	−64.14	10	92 (16–221)	63 (28–141)
	AIS	−64.25	−65.24	14	126 (57–306)	62 (21–190)
juvenile	CAS	−60.80	−62.46	5	30 (12–76)	43 (23–113)
	CVA	−60.98	−64.14	5	28 (9–85)	63 (30–112)

We processed raw location estimates by removing four adult deployments that were tracked less than 7 days, and all erroneous location estimates indicated by ‘Z’ quality codes or unspecified ellipse errors. Next, we applied a speed filter [26] assuming a conservative swim speed of 2.5 m/s. Remaining tracks were smoothed with a state-space model [27] using the R [28] package ‘crawl’ [29]. Model fits were used to generate 100 alternative tracks for each deployment, with locations estimated every 2 h. Alternative tracks were pooled and mapped to hexagonal polygons with centroids spaced 15 km apart (area ≈ 87 km^2^) to estimate habitat utilization distributions (HUDs) using the R package ‘crawlr’ [30]. This spatial scale approximates daily foraging ranges by gentoo penguins during the breeding season [25].

We used several physical variables to compare the habitats used by birds from different colonies. We extracted bottom depths along each track from the ETOPO1 dataset [31]. We estimated distance to the nearest point of land using the ‘wrld_simpl’ database in the R [28] package ‘maptools’ [32]. To examine near real-time experience of sea-surface temperatures (SST) and sea ice concentrations (SIC, expressed as per cent cover), we matched raw position estimates with daily SSTs from the multi-scale ultra-high-resolution SST data resolved on a 1 km grid [33] and daily SICs from the EUMETSAT Ocean and Sea Ice Satellite Application Facility, projected from a native 10 km grid to a 1 km grid [33].

We used the Tukey honest significant difference (HSD) to identify colony-level differences in movement and linear mixed-effects models using the R [28] package ‘lme4’ [34] to identify colony-level differences in physical habitat variables. We fitted separate models for each physical variable and included month as a fixed effect in all models to account for potential seasonal trends. Individuals were treated as random effects.

## 3. Results

**Figure 1.**
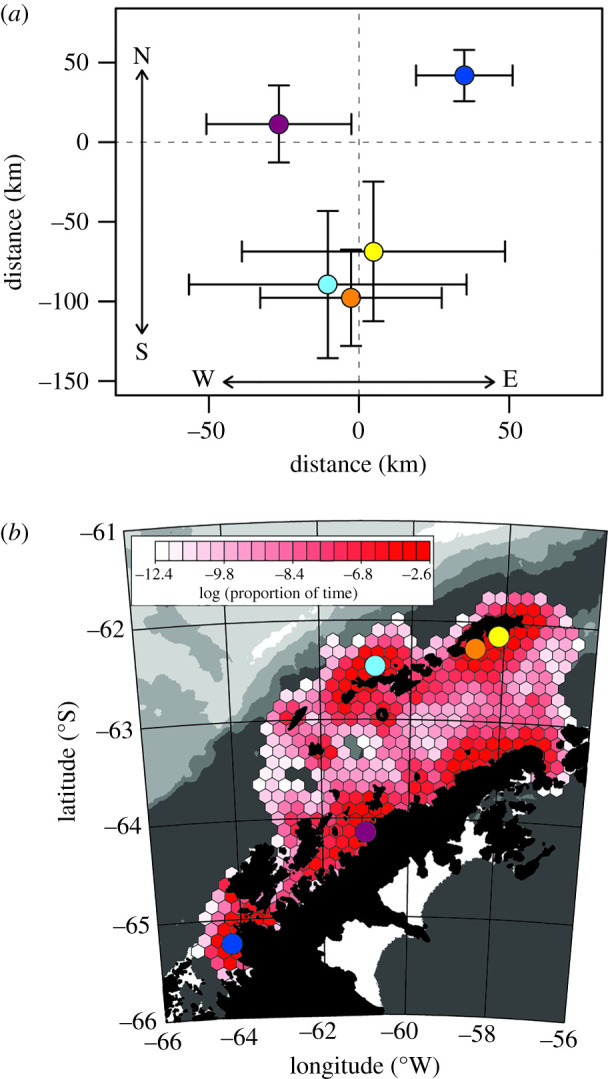
(*a*) Mean and 95% confidence intervals for positional shifts for birds from the Argentine Islands (dark blue), Cierva Cove (purple), Cape Shirreff (light blue), Stranger Point (orange) and Lions Rump (yellow). (*b*) HUDs for all tracked birds. Colony locations are indicated with circles coloured to match panel (*a*).

Positions (*N* = 29 119) of fledgling and adult penguins were, respectively, reported for an average of 29 days (range: 9–86 days; table 1) and 100 days (range: 16–306 days). Maximum distances from tagging sites varied by colony (table 1). Gentoo penguins originating from the northern edge of their range in the WAP dispersed farther and with significantly greater shifts to the south (Tukey HSD *F*_4,66_ = 12.8, *p* < 0.01) than adults tagged at colonies further southwest (figure 1*a*). Longitudinal movements were not different across colonies (figure 1*a*, Tukey HSD *F*_4,66_ = 1.9, *p* = 0.11). Given high overlap of fledglings and adults (electronic supplementary material, figure S2), all tracks were pooled for further analyses.

**Figure 2.**
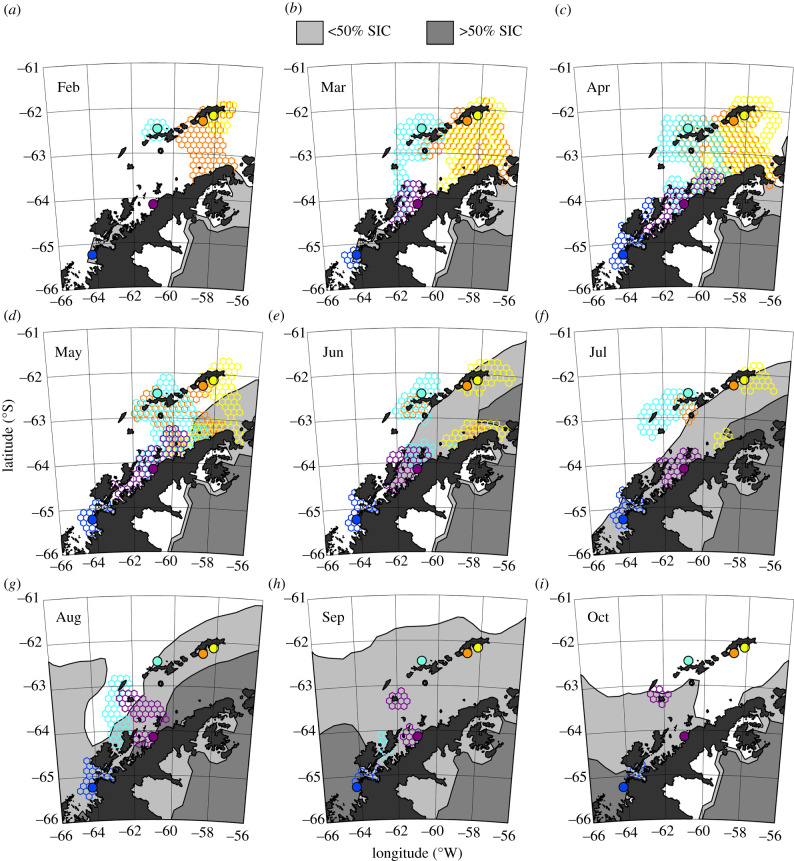
Monthly extents of colony-specific HUDs overlaid on monthly SIC. Colony locations are identified by filled circles. Colony locations and their HUDs are coloured as in figure 1.

The physical habitats encountered during winter were largely similar across colonies (electronic supplementary material, figure S3). The model-predicted distance from shore averaged 6.4 km with little variation among colonies (table 2). Near shore areas corresponded to shallow habitats that averaged 42 m deep across all colonies (table 2), noting that birds from Cape Shirreff and the Argentine Islands had the shallowest habitats. Birds from Cape Shirreff, with a more northern distribution for much of the winter, typically encountered warmer water than birds from other colonies (table 2). Responses to the distribution of SIC were not colony-specific when ice was encountered (table 2), and all birds usually occupied ice-free waters (figure 2).

**Table 2.** Mixed-effect model predictions for the fixed-effects (95% CI) of colony origin on distance from shore, depth, sea-surface temperatures (SST) and sea ice concentrations (SIC) for all tracked penguins.

colony	distance to shore (km)	depth (m)	SST (°C)	SIC (%)
LRP	7.6 (3.9–11.5)	52 (32–86)	1.03 (0.74–1.32)	39 (25–51)
SPS	6.0 (2.1–9.8)	67 (41–111)	1.0 (0.7–1.29)	38 (29–47)
CAS	6.3 (2.4–10.1)	24 (14–40)	1.62 (1.33–1.91)	34 (22–46)
CVA	4.3 (0.5–8.1)	53 (32–88)	0.83 (0.54–1.12)	37 (24–49)
AIS	7.9 (4.2–11.8)	18 (11–30)	1.06 (0.77–1.35)	45 (33–57)

The aggregate HUD for all tracks during winter (figure 1*b*) demonstrates an affinity for coastal areas. Winter HUDs were concentrated near tagging sites, around islands in the Bransfield Strait, and along the entire margin of the WAP from the Argentine Islands to the tip of the Peninsula (figure 1*b*). Overlap of colony-level HUDs (figure 2) was common. This was particularly evident for birds from Stranger Point and Lions Rump, which overlapped extensively around King George/25 de Mayo Island and along the northern tip of the Peninsula from 59° W to 57° W. Similarly, birds from Cape Shireff and Cierva Cove overlapped extensively along the central WAP from 63° W to 59° W. The HUDs for gentoo penguins from the Argentine Islands, the most southern colony, were largely isolated from other colonies.

Differences in colony-level movements from February to April occurred prior to the presence of sea ice near any colony (figure 2), demonstrating that sea ice was not the main driver of colony-specific, over-winter dispersal patterns. Nonetheless, the evolution of dense sea ice (SIC > 50%) did affect the distribution of the birds during winter. For example, the HUDs of birds from Lions Rump contracted between June and July when high SICs blanketed the eastern Bransfield Strait. Likewise, birds from the Argentine Islands shifted northeast into the Gerlache Strait coincident with the expansion of dense sea ice in waters south of Anvers Island from August to October (figure 2). However, SIC < 50% did not preclude gentoo occupation of those areas (figure 2).

## 4. Discussion

We report clear latitudinal gradients in winter movements of gentoo penguins from five colonies of varying population sizes and trends along the WAP. Dispersal distances were larger for birds from northern colonies than from southern colonies. The HUDs of birds from different colonies overlapped in the relatively shallow, ice-free coastal margin of the WAP. Of particular importance were the coastal regions of the Antarctic Peninsula from the Argentine Islands to the tip of the Antarctic Peninsula. Our multi-colony tracking study demonstrates that, in the WAP, the coastal habitat essential to breeding gentoo penguins during summer is similarly critical during winter. The observed gradient in movement patterns suggests that birds from northern colonies are immigrant sources for the current range expansion of this species.

Marine top predators are often expected to change foraging behaviours, movement patterns and at-sea distributions [35] in response to climate-driven changes in prey distribution [9], rather than to direct changes in their physical environment. In the WAP, however, sea ice dynamics can fundamentally alter the availability of foraging habitats. As an ‘ice-intolerant’ species [36], we expected the movement patterns of gentoo penguins to be driven by avoidance of developing sea ice. However, all long-distance dispersal observed here was initiated before the presence of sea ice at study colonies. Indeed, winter movements of gentoo penguins in the Falkland/Malvinas Islands, where sea ice does not exist, were even farther than observed in the WAP [37]. Thus, sea ice is not the main driver of differences in colony-specific dispersal patterns among gentoo penguins. As sea ice extent and duration in the WAP are expected to decline under most climate-change scenarios [38], the observed latitudinal gradients in movement and the affiliation of gentoo penguins with coastal regions along the WAP may be expected in the future.

Dispersal of seabirds from breeding sites to winter foraging areas must be driven by reliable availability of prey. Gentoo penguins typically exhibit benthic and pelagic foraging dives (less than 150 m depths) in coastal regions [20] with diets of crustaceans (mainly Antarctic krill), other invertebrates and fishes that can vary by location [36]. Within the WAP, shorter dispersal ranges of gentoos from southern colonies relative to northern colonies suggest greater food availability along the margins of the Antarctic Peninsula relative to the south Shetland Islands during winter. The continental shelves of the WAP, over which the HUDs were concentrated, are known to harbour high krill densities during winter (e.g. [39,40]), consistent with expectations of a southward contraction of krill distributions over larger scales [41]. These shallow, coastal areas also provide gentoo penguins with access to benthic and demersal resources, which are suspected to be important components of their winter diets [42].

The tracking data reported here suggest source populations for the ongoing range expansion of gentoo penguins in the WAP. Genetic analysis indicates that basin-scale dispersal and colonization events are rare for this species, and the Polar Front is an effective boundary between sub-Antarctic and Antarctic populations [23,43]. At smaller spatial scales, gentoo penguins are well-known colonizers of new breeding territory and quickly take advantage of ice-free breeding space [36]. Recent colonization events have been attributed to emigration from colonies at the southwestern edge of this species' range [12,44]. However, longer distance movements of birds from northern colonies suggest that rare dispersal events to the south could be instigated by birds from northern colonies. Such a leap-frog migration strategy has been reported for other seabirds [45]. Differential movement patterns of birds from different breeding colonies may be driven by variation in prey availability at breeding colonies but shared foraging habitat preferences that favour gradients in directed movement [45,46]. While we cannot test this hypothesis directly, the observed latitudinal gradient in the movement of gentoo penguins is consistent with a leap-frog strategy. In particular, larger scale movements during the winter provide an opportunity to prospect new haul-out sites and nesting areas that would support colonization events.

The effects of ongoing climate change in the WAP region are difficult to predict [47]. However, continued reduction in SIC during winter may be advantageous for coastal predators and an increase in the availability of ice-free foraging habitats may facilitate southward expansion in the breeding range of gentoo penguins. Nonetheless, such expectations may be tempered by increases in the biomass of salps [48] with concomitant declines in krill biomass due to recruitment failures [41], local increases in the abundances of cetaceans (potential competitors with gentoo penguins for food) [49] and continued growth of the krill fishing industry [6,8]. Our study suggests that, in the WAP, a latitudinal gradient in the movement of gentoo penguins during winter might be a key to the dynamic of how gentoo populations cope with large-scale changes in the ecosystem.

## Supplementary Material

Figures S1 - S3

## Data accessibility

Data for this article are available from the Dryad Digital Repository at: https://doi.org/10.5061/dryad.5x69p8d20 [50].
