# Supplementary material for: Coastal regions of the northern Antarctic Peninsula are key for gentoo populations
Source: Biol Lett. 2021 Jan 27;17(1):20200708. doi: 10.1098/rsbl.2020.0708 (PMC7876601; doi:10.1098/rsbl.2020.0708)
Supplement: Figures S1 - S3 [file rsbl20200708supp1.docx]

**Electronic Supplemental Material**

**Biology Letters**

**Coastal regions of the northern Antarctic Peninsula are key for gentoo populations**

Korczak-Abshire Malgorzata^1*^, Hinke Jefferson T.^2^, Milinevsky Gennadi ^3,4^, Juáres Mariana A.^5,6^, Watters George M.^2^

^1^ Institute of Biochemistry and Biophysics, Polish Academy of Sciences, 02-106 Warsaw, Poland

^2^ Antarctic Ecosystem Research Division, Southwest Fisheries Science Center, National Marine

Fisheries Service, National Oceanic and Atmospheric Administration, La Jolla, California,

92037, USA

^3^ Department of Atmospheric Physics and Geospace, National Antarctic Scientific Center of Ukraine,

Kyiv, 01601, Ukraine

^4^ Physics Faculty, Taras Shevchenko National University of Kyiv, Kyiv, 01033, Ukraine

^5^ Departamento Biología de Predadores Tope, Instituto Antártico Argentino, San Martín, Buenos

Aires, B1650CSP, Argentina

^6^ National Scientific and Technical Research Council (CONICET), Ciudad Autónoma de Buenos

Aires, C1425FQB, Argentina

* Author for correspondence: Malgorzata Korczak-Abshire · mka@ibb.waw.pl

Contents:

**Figures S1, S2** and **S3**

Data for article and electronic supplemental material are available in the Dryad Digital Repository at: https://doi.org/10.5061/dryad.5x69p8d20

**Figure S1.** Distribution of gentoo penguin (*Pygoscelis papua*) breeding sites within the western Antarctic Peninsula region and CCAMLR Statistical Area No. 48.1 (red line); (*a*) current breeding sites with seven newly established colonies (data source: Mapping Application for Penguin Populations and Projected Dynamics - MAPPPD, see Humphries *et al*. 2017); (*b*) active sites and the estimated numbers of breeding pairs from a census in 2017 or the latest one before 2017 available at MAPPPD when applicable; (*c*) population trends (1990-2017) at five investigated breeding sites: Lions Rump (LRP) and Stranger Point (SPS) on King George Island, Cape Sherriff (CAS) on Livingston Island, the Argentine Islands (AIS) within Wilhelm Archipelago, and Cierva Cove (CVA) on the Antarctic Peninsula [Dykyy et al. 2019, González-Zevallos *et al*. 2013, Hinke *et al*. 2017, Juáres *et al*. 2020, Korczak-Abshire *et al*. 2013]. Produced using data from the SCAR Antarctic Digital Database and CCAMLR GIS web site.


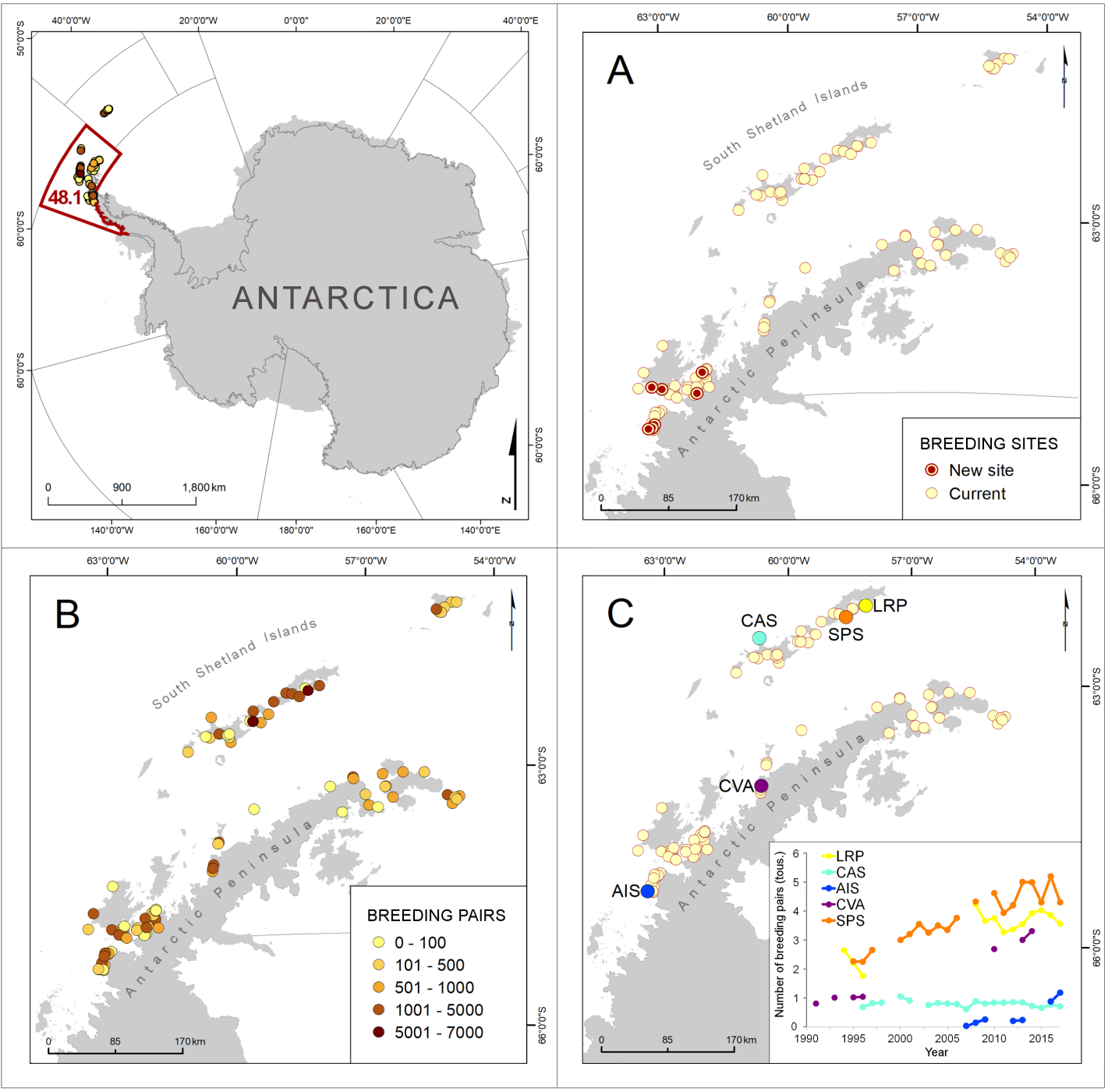


**Figure S2**. Location estimates for adult and fledgling gentoo penguins tracked from five investigated breeding colonies (see Figure S1c) in the western Antarctic Peninsula region during the austral winter of 2017 (Dryad Digital Repository, see Korczak-Abshire *et al.* 2020).


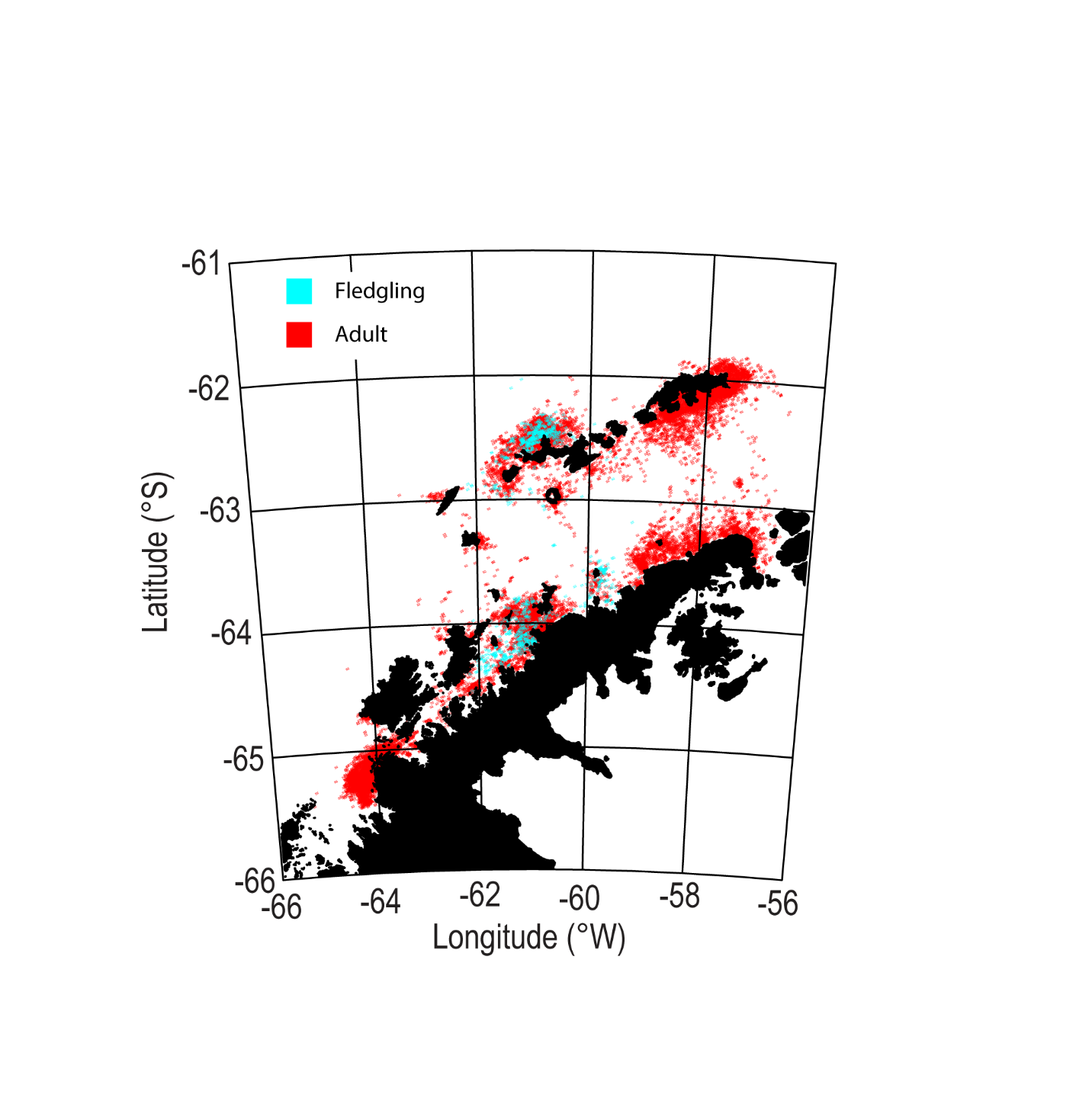


**Figure S3**. Monthly distributions of physical habitat variables encountered by all-aged gentoo penguins originating from the five investigated colonies. Only data for February to October are displayed in the main manuscript (Dryad Digital Repository, see Korczak-Abshire *et al.* 2020).


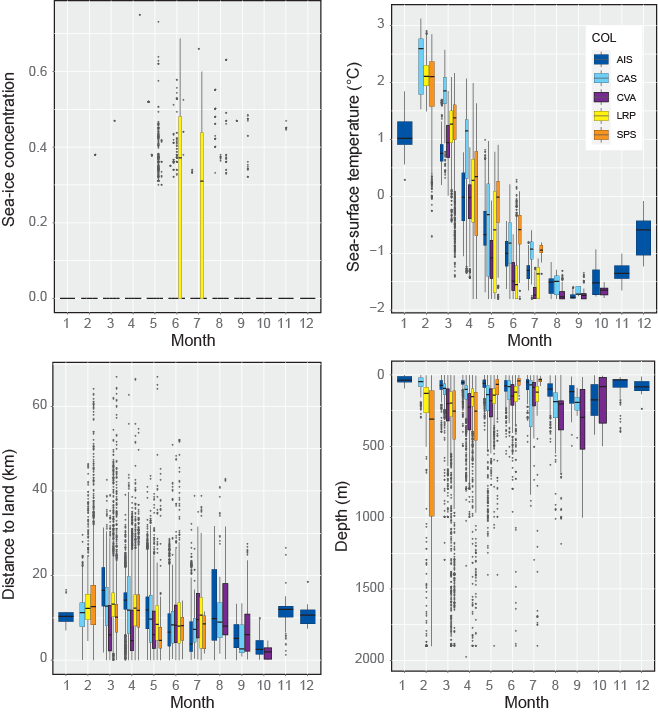


**References**

Dykyy І V. *et al.* 2018 Features of Chronology and Breeding Success of *Pygoscelis papua* and *P. аdeliae* (Spheniscidae) Penguins in the Wilhelm Archipelago (CCAMLR Subarea 48.1). *Ukr. Antarct. J.* **1**, 130–147. (doi:10.33275/1727-7485.1(17).2018.39)

González-Zevallos D, Santos MM, Rombolá EF, Juáres MA, Coria NR. 2013 Abundance and breeding distribution of seabirds in the northern part of the Danco Coast, Antarctic Peninsula. *Polar Res.* **32**, 11133. (doi: 10.3402/polar.v32i0.11133)

Hinke JT, Cossio AM, Goebel ME, Reiss CS, Trivelpiece WZ, Watters GM. 2017 Identifying Risk: Concurrent overlap of the antarctic krill fishery with krill-dependent predators in the Scotia Sea. *PLoS One* **12**, 1–24. (doi:10.1371/journal.pone.0170132)

Humphries GRW, Naveen R, Schwaller M, Che-Castaldo C, McDowall P, Schrimpf M, Lynch HJ. 2017 Mapping Application for Penguin Populations and Projected Dynamics (MAPPPD): Data and tools for dynamic management and decision support. *Polar Rec. (Gr. Brit).* **53**, 160–166. (doi:10.1017/S0032247417000055)

Juáres MA, Casaux R, Negrete J, Rios A, Castillo M, Coria NR, Santos MM. 2020 Update of the population size and breeding performance of gentoo penguins ( *Pygoscelis papua* ) at Stranger Point / Cabo Funes , South Shetland Islands. *Polar Biol.* **43**, 123–129. (doi:10.1007/s00300-019-02614-0)

Korczak-Abshire M, Wegrzyn M, Angiel PJ, Lisowska M. 2013 Pygoscelid penguins breeding distribution and population trends at Lions Rump rookery, King George Island. *Polish Polar Res.* **34**, 87–99. (doi: 10.2478/popore−2013−0002)

Korczak-Abshire, M, Hinke JT, Milinevsky G, Juáres MA, Wattes GM. 2020 Datasets supporting: Coastal regions of the northern Antarctic Peninsula are key for gentoo populations. Dryad Digital Repository https://doi.org/10.5061/dryad.5x69p8d20
